# The Future of Precision Prevention for Advanced Melanoma

**DOI:** 10.3389/fmed.2021.818096

**Published:** 2022-01-17

**Authors:** Katie J. Lee, Brigid Betz-Stablein, Mitchell S. Stark, Monika Janda, Aideen M. McInerney-Leo, Liam J. Caffery, Nicole Gillespie, Tatiane Yanes, H. Peter Soyer

**Affiliations:** ^1^Dermatology Research Centre, The University of Queensland Diamantina Institute, The University of Queensland, Brisbane, QLD, Australia; ^2^Centre for Health Services Research, School of Medicine, The University of Queensland, Brisbane, QLD, Australia; ^3^The University of Queensland Business School, Faculty of Business, Economics and Law, The University of Queensland, Brisbane, QLD, Australia; ^4^Department of Dermatology, Princess Alexandra Hospital, Brisbane, QLD, Australia

**Keywords:** melanoma, prevention, artificial intelligence, genomics, risk stratification

## Abstract

Precision prevention of advanced melanoma is fast becoming a realistic prospect, with personalized, holistic risk stratification allowing patients to be directed to an appropriate level of surveillance, ranging from skin self-examinations to regular total body photography with sequential digital dermoscopic imaging. This approach aims to address both underdiagnosis (a missed or delayed melanoma diagnosis) and overdiagnosis (the diagnosis and treatment of indolent lesions that would not have caused a problem). Holistic risk stratification considers several types of melanoma risk factors: clinical phenotype, comprehensive imaging-based phenotype, familial and polygenic risks. Artificial intelligence computer-aided diagnostics combines these risk factors to produce a personalized risk score, and can also assist in assessing the digital and molecular markers of individual lesions. However, to ensure uptake and efficient use of AI systems, researchers will need to carefully consider how best to incorporate privacy and standardization requirements, and above all address consumer trust concerns.

## Introduction

Clinician-led skin examinations with dermoscopy are the mainstay of melanoma detection, with unaided (“naked-eye”) examinations alone now considered insufficient (1). Dermoscopy requires some training to use effectively, but significantly improves the specificity of diagnosis (2). Increasingly, high-risk patients are managed with total body photography and sequential digital dermoscopic imaging, (3) allowing clinicians to monitor for changes in melanocytic naevi (moles) over time; this is particularly useful for patients with many atypical naevi (commonly called dysplastic naevi) (4). Clinician-led screening of high-risk patients is associated with earlier detection and a better prognosis, but imprecise diagnosis continues to have a major impact on patients and the health system (5).

Underdiagnosis, a missed or delayed melanoma diagnosis, leading to untreated or improperly treated disease, is a familiar problem to clinicians. This is particularly undesirable in melanoma, where a correct early diagnosis often allows successful treatment with a simple excision, while advanced melanoma treatment is expensive and associated with a poorer prognosis and undesirable side effects of treatment (6, 7). Medico-legal fears also incline clinicians to excise rather than monitor a suspicious lesion and patients often express a preference for an early excision (5, 8).

Overdiagnosis is a less well-known but increasingly recognized problem, defined as detecting true cancers that are so slow-growing (indolent) that they would not cause a problem in the patient's lifetime (9). Overdiagnosis is a common observation in many cancers such as thyroid cancer and breast cancer (9) and, remarkably, it is estimated that up to 58% of melanomas in Australia are overdiagnosed (10). These indolent cancers are currently indistinguishable from melanomas with invasive potential, so they are typically also excised for patient and clinician peace of mind. These potentially avoidable excisions add an extra burden to the health care system and increase a patient's risk of scarring, infection, and other adverse events (5). In addition, the diagnosis of melanoma, even *in-situ* melanoma, can incur psychological distress (11). The same techniques that enable early detection of thin but potentially invasive melanomas also appear to increase detection of indolent melanomas. It is critical that we learn to distinguish melanoma interventions that actually benefit patients long-term from those that promote overdiagnosis (12), and to differentiate between slow-growing and potentially invasive melanomas (13).

Precision prevention of advanced melanoma has been proposed to address these problems. It consists of first stratifying patients into an appropriate level of surveillance with personalized risk scores, which combine demographic, phenotypic and genetic risk factors, and then customizing their screening requirements accordingly. By using a low-intensity surveillance regimen for people identified to be at low-risk, the likelihood of overdiagnosis is decreased. Low-risk surveillance may consist of education to promote self-skin examination, thereby bringing new or changing lesions to primary care providers. In contrast, high and ultra-high risk patients (those who have multiple risk factors or have already had one or more melanomas, respectively) could potentially benefit from more intensive surveillance by clinicians using total body imaging and sequential digital dermoscopy that can detect early changes of emerging melanomas, especially in patients with multiple and/or atypical naevi, where a diagnosis without photographic documentation may be difficult. For these patients, it may also soon become possible to use a combination of molecular and digital biomarkers, collected through non-invasive or minimally-invasive techniques, to assess individual lesions for their likelihood to be a true melanoma or an aggressive melanoma.

## Risk Stratification

**Figure 1:**
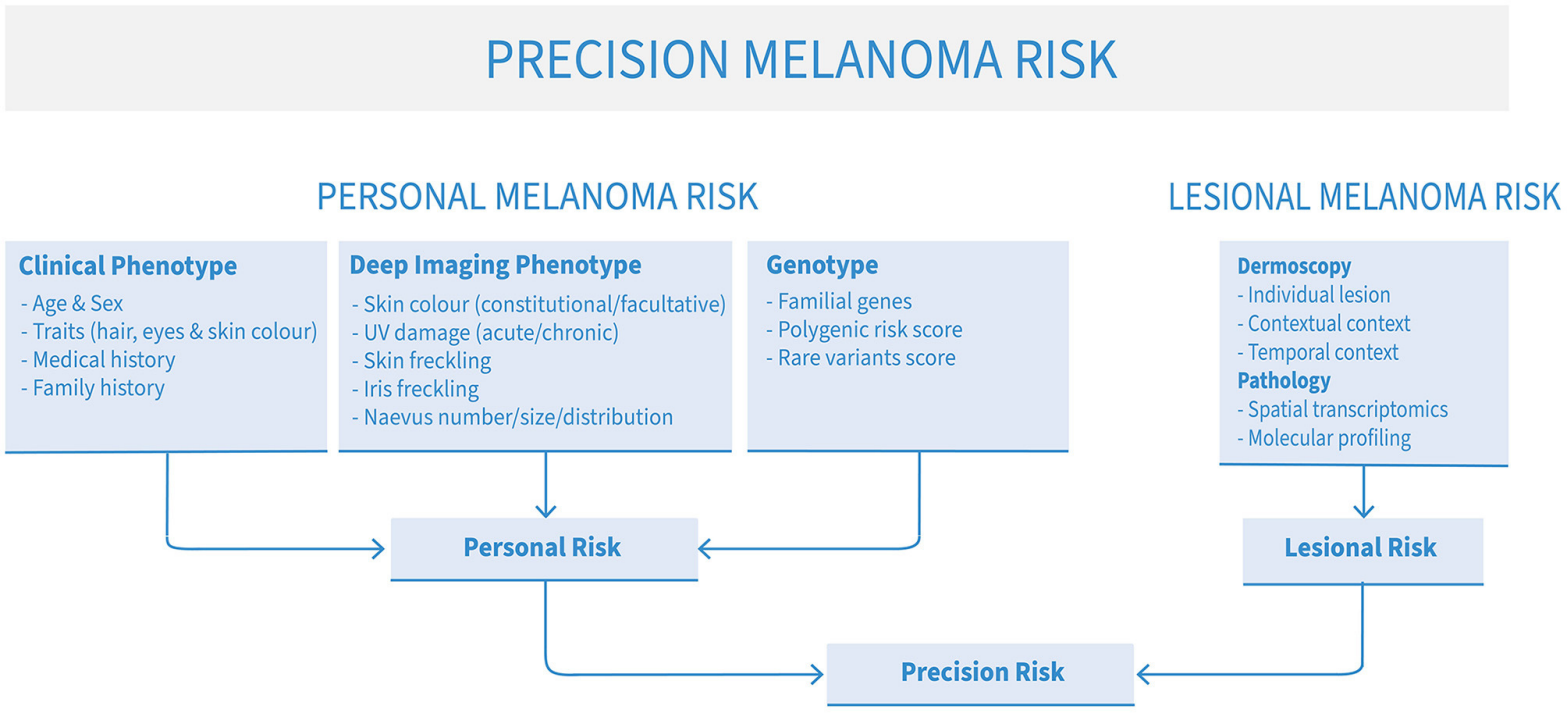
The elements of precision melanoma risk scores for risk stratification and precision prevention of advanced melanoma.

Several types of risk can be assessed to approximate a patient's risk of developing melanoma: clinical phenotype, comprehensive imaging phenotype of sub-clinical factors (“deep image-based phenotype”), familial and polygenic risks. For best results, however, these assessments can be combined to produce a more nuanced, personalized holistic risk score (Figure 1). Clinical risk comprises readily-observable data such as age, sex, pigmentation traits of hair, eye and skin color, number of naevi, personal and family melanoma history. This kind of data is commonly used by clinicians in *ad-hoc* assessments of melanoma risk. However, deep image-based phenotyping, polygenic and familial genetic risk are newer approaches that are slowly being integrated in clinical use. In addition, the particular risk profile of each patient may direct clinicians to be on the lookout for particular types of melanoma, such as lentigo maligna melanoma in older patients with severe chronic UV damage, many solar lentigines and a history of basal or squamous cell carcinomas (14), or amelanotic lesions in patients with mutations in the albinism pathway (15).

### Clinical Phenotype

Clinical phenotype, already used in an *ad-hoc* way by many clinicians to assess patients' melanoma risk, is an important inclusion in a holistic risk score. Age, sex, pigmentation traits of hair, eye and skin color, and number of large naevi are well-known melanoma risk markers. Non-melanoma skin cancers, as well as multi-cancer syndromes such as Li-Fraumeni syndrome (16) further add to risk estimates. Finally, prior and ongoing medical treatment, such as immunosuppressive treatment or PUVA, while relatively rare compared to other clinical phenotype markers, may also be included, although their link with basal and squamous cell carcinomas are much stronger than melanoma (17, 18).

### Deep Image-Based Phenotype

Deep image-based phenotyping is the concept of creating an automated and objective assessment of phenotypic melanoma risk factors directly from total body imaging. Such measures include constitutional and facultative skin color, naevus phenotype, freckling phenotype and UV damage phenotype; these sub-clinical factors are known melanoma risk indicators (19).

It is well known that those with fairer skin tones are at higher risk of developing melanoma; however in those with darker skin tones, melanoma is often diagnosed later and has higher rates of mortality (20). While skin color is a continuous measure, it is often categorized for ease of assessment. The Fitzpatrick skin type is commonly used and is calculated based on pigmentation traits and self-report of the skin's reaction to the sun. While easy to calculate, it relies on the subjective assessment of the individual and/or healthcare provider and is a poorer proxy in those with darker skin tones (21). The individual topography angle (ITA) maps color onto a 2-dimentional space using the CIE L^*^a^*^b^*^, with gold standard measures achieved using a spectrophotometer or colorimeter. However, such measures can also be extracted directly from digital images, (22). eliminating the need for specialist equipment and removing subjectivity.

A high total body naevus count has long been known to be a strong melanoma risk factor. A lack of adoption of a standard protocol for counting naevi has resulted in little consistency across studies, with variations in who counts them (clinicians, researchers) and the size counted (>2 mm, >3 mm, >5 mm) (23, 24). In addition naevus counts are time-consuming and therefore studies often rely on self-report, which tends to have low agreement with experts, and can lead to misclassification of risk (25). As part of the deep image-based phenotype, automated objective naevus counts can be obtained using convolutional neural networks applied to 3D total body photography (26).

UV exposure is the primary environmental risk factor, but quantifying an individual's chronic exposure level has been difficult, largely relying on self-report as to time in the sun and protective strategies used. We have shown that photo-numeric scales can be accurately used to grade sun damage across all body sites (27), and we are currently automating this process with convolutional neural networks. Freckling is also a well-known risk factor, (28). indicating UV exposure interacting with defects in pigmentation genes such as *MC1R*. Similar to the methods applied to assess UV damage, an automated measure of freckling density is also being developed by our group.

### Familial Melanoma Genetics and Polygenic Risk Scores

Twin studies have estimated melanoma heritability to be 55% (29), and first-degree relatives of an affected individual have a two-fold increased risk of developing melanoma in their lifetime (30). Approximately 10% of melanoma is familial, but only 20% of melanoma-prone families will carry a mutation in a known melanoma gene (31). In 90% of positive cases, the mutation occurs in *CDKN2A*, with mutations being more rarely identified in *CDK4, BAP1, BRCA1, BRCA2, MITF, PTEN, TERT, POT1, POLE, TERF2IP, ACD, RB1* and *TP53*. (16, 31–34). Individuals with mutations in *CDKN2A* have a 52% average lifetime risk of developing melanoma, with an increased risk of developing multiple melanomas, and a higher probability of being diagnosed at an earlier age (35) *CDKN2A* mutation carriers' lifetime melanoma risk is further increased if they also carry common red hair color variants in *MC1R* (36). Recent systematic reviews have found that *CDKN2A* testing is associated with minimal, if any distress (37) and some positive impacts on primary and secondary preventative behaviors (38).

Though melanoma risks are significant in familial melanoma cases, they account for a relatively small portion of individuals diagnosed with melanoma annually. A meta-analysis of genome-wide association studies comparing hundreds of thousands of individuals with and without a personal history of melanoma has found 68 single nucleotide polymorphisms (SNPs) in 54 locations across the genome implicated in melanoma risk (39). Each of these SNPs is associated with an individual risk ratio or odds ratio. These weighted risks can be summed to generate a single, cumulative disease-specific polygenic risk score (PRS). These have been created for multiple cancers, cardiovascular disease and mental illnesses with the goals of population risk stratification, risk refinement in high-risk families and informing clinical management (40). Early studies in diverse disease groups show that communication of this risk information is not associated with undue psychological sequelae or adverse health behaviors (41). In keeping with familial melanoma testing, initial studies communicating melanoma PRS in the general population show no impact on psychological distress and a positive improvement in some primary preventative behaviors (42).

## Individual Lesion Assessment

### Digital Markers

Since the seminal paper was published on the topic (43), Convolutional Neural Networks (CNNs) have been applied to individual dermoscopic lesion images, with research showing that automated algorithms can, in most cases, classify lesions with higher accuracy than dermatologists (44). Human-computer collaboration has been shown to further improve accuracy (45). Several commercial software offer dermoscopic lesion classification and also provide a malignancy risk score. Automated algorithms are now being extended to closer represent the clinical environment, incorporating within-patient context by providing the algorithm multiple images per timepoint (46). It is now possible for automated algorithms to incorporate longitudinal series of dermoscopic images, with initial results indicating the algorithm is able to detect melanoma earlier than clinicians while still avoiding overdiagnosis (47). Additionally, such techniques are being applied to clinical images to identify suspicious naevi (48). Image processing methods are also being used by software such as Canfield Scientific Inc (Parsippany, NJ, USA) VAM module, which can identify individual lesions from 3D total body photography, and provide lesion metrics such as diameter, hue and asymmetry (26). Additionally, through image processing and markerless tracking technology, lesions can be tracked over time to monitor changes in color, size and shape.

### Spatial Transcriptomics and Molecular Profiling

Techniques for molecular analysis of DNA and RNA have rapidly evolved in the past few years, leading to efforts to develop a refined and integrated molecular signature that could reliably detect melanoma using a minimally-invasive technique, such as a micro-biopsy or tape-stripping device (49). This aims to allow analysis of suspicious melanocytic lesions without requiring a full sized biopsy, particularly useful for patients with high numbers of atypical lesions that meet the criteria for excision. Each specimen would be analyzed for precise hallmarks of melanoma, and the lesion would only be excised if a positive signal was identified.

Current testing for *BRAF, NRAS, HRAS* and *cKIT* mutation have been recognized as useful clinical markers for advanced melanoma therapy decision-making, but the prevalence of these mutations in benign melanocytic lesions makes them impractical for early detection purposes (50). Gene expression profiling (GEP), using a panel of genes known to be differentially expressed between benign and malignant melanocytic lesions, may become a useful technique here; however commercially available GEP panels require further evaluation against standard-of-care clinicopathologic risk markers to verify that they add value over the current clinical, genetic and phenotypic risk profile (51).

With the advances of deep sequencing technologies, it is now routine to survey the whole genome and transcriptome from a fresh tissue biopsy. These powerful tools have fast tacked the discovery of drug targets for cancer treatment (52), tumor mutational load for prediction of immunotherapy outcome (53), and importantly the discovery of novel and interacting signaling pathways to greater understand cancer progression (54).

While previously these analyses were conducted on all cells present in the tissue (“bulk” sequencing analysis), single-cell technologies are now available which permit discrete molecular profiling of each cell type present in the tissue biopsy (55). These tools combined with the deep sequencing technologies have enabled precise gene expression analysis, thus allowing cell-type (or cell state, e.g., malignant) specific profiles to be discovered to empower progression biomarker discovery (56).

Spatial profiling, including spatial transcriptomics, is another emerging technology which will revolutionize our understanding of lesion heterogeneity. These technologies allow for the analysis of whole transcriptomes, spatially resolved to defined regions of interest within histopathology tissue sections, allowing a comparison of histopathologically-identifiable melanoma structures and their molecular profiles (57, 58).

These cutting-edge tools currently determine the complete molecular profile of the whole tissue from a complete excision or punch biopsy. Their integration into microbiopsy, tape-stripping or other minimally-invasive devices will be critical for delivering individual lesion molecular assessment to the clinic.

## Considerations for Implementation

### Consumer Trust in AI Computer-Aided Diagnostics

Central to the acceptance and use of technology-aided diagnostics is consumer and clinician trust. Technology-aided diagnostics and teledermoscopy services bring many benefits for consumers, such as convenience, reduced travel time, fewer unnecessary referral for benign lesions, potential costs savings, (59). and improved triage and management (60, 61), but barriers to consumer trust and uptake include privacy and confidentiality concerns, diagnostic confidence, and concerns around inadequate patient-clinician interaction (61, 62). When skin self-examination is conducted using teledermoscopy, additional barriers include technological difficulties and the challenge of conducting whole body skin self-examination. A recent study of teledermoscopy consumers revealed modest trust levels and decreased acceptance following experience with using the technology, but also a willingness to use it again in future (63).

Trust issues are likely to be exacerbated with the inclusion of artificial intelligence (AI) in diagnostics, despite its potential ability to increase diagnostic accuracy (45), due to the black-box nature of many AI algorithims, which do not explicitly show users how the algorithm came to its conclusion. A recent representative study of over 6,000 people across five western countries indicates only 37% of people are willing to trust AI-enabled health diagnostic services (64). The exact way AI technology should be used to support the early diagnosis of melanoma is also not yet clear, with some proposing that AI should triage lesions so that the workload of clinicians would be reduced, while others propose AI should provide a second opinion so that clinicians could reassess lesions where the AI diagnosis differs from their own (45, 65).

### Standardization

Another barrier to technological uptake in the clinic, particularly AI uptake, is lack of standardization (66). Digital Image Communication in Medicine (DICOM) is the standard in medical imaging (67). DICOM provides a standardized way to encode and store medical images and their associated metadata, but more importantly DICOM is an interoperability standard that facilitates the sharing of medical images and associated data both within and between organizations.

The first version of DICOM was published in 1985. It has been evolving in some medical image-producing specialities (e.g., radiology and cardiology) since then and now enjoys ubiquitous use (68). However, it was not until 2020 that the first dermatology-specific extension to the DICOM standard was published (69). Until recently, (68). dermatology imaging largely consisted of clinical images acquired on commercial, off-the-shelf cameras and smart devices. The need for standardization and the adoption of DICOM for dermatology has been driven by a number of factors including the clinical use of advanced imaging modalities (e.g., total body photography, confocal microscopy), the use of sequential dermoscopic imaging, teledermatology, and the potential of AI.

The adoption of standards for dermatology imaging can improve AI workflows by encoding derived objects (e.g., secondary images, visual explainability maps, AI algorithm output) and the efficient curation of multi-institutional datasets for machine learning training, testing, and validation (70). The use of DICOM for the management of dermatological images will not guarantee effective clinical translation of AI in dermatology but may address important technological and implementation challenges (70).

### Privacy

Addressing privacy in dermatology imaging is a further very relevant implementation consideration. The use of dermatological imaging and AI in dermatology is currently impeded by lack of guidance for clinicians and researchers on the acceptable use of the images. Further, patients may not fully understand the possible privacy consequences of interacting with these technologies. There are dermatology-specific issues such as nudity in total body photography and difficulty in de-identifying data for secondary use due to the patient being visually identifiable that are not addressed in existing health privacy frameworks (68).

## Conclusion

Precision prevention of advanced melanoma is fast becoming a realistic prospect, with remaining obstacles well-defined and under investigation by many researchers. A major challenge is promoting consumer trust in these emerging technologies, along with prioritizing privacy and standardizing image collection to allow AI algorithms to work effectively. However, if we are able to meet these challenges, risk stratification, using clinical and subclinical, deep image-based phenotype, familial and polygenic risk factors, combined with increasingly sophisticated assessment of digital and molecular markers, promises to continue to improve early melanoma detection and surveillance for those at ultra-high risk while minimizing overdiagnosis.

## Data Availability Statement

The original contributions presented in the study are included in the article/supplementary material, further inquiries can be directed to the corresponding author.

## Author Contributions

HS and KL contributed to conception of the paper. KL wrote the first draft of the manuscript. BB-S, AM-L, LC, MJ, NG, MS, TY, and HS wrote sections of the manuscript. All authors contributed to manuscript revision, read, and approved the submitted version.

## Funding

HS holds an NHMRC MRFF Next Generation Clinical Researchers Program Practitioner Fellowship (APP1137127). MS was supported by the Marchant Charitable Foundation. AM-L was funded by a National Health and Medical Research Council (NHMRC) Early Career Fellowship (ID 1158111). NG was partially funded by the industry grant KPMG Chair in Organizational Trust (RM2018001776). This research was conducted with the support of the NHMRC Center of Research Excellence in Skin Imaging and Precision Diagnosis (APP2006551) and the ACRF Australian Center of Excellence in Melanoma Imaging and Diagnosis.

## Conflict of Interest

HS is a shareholder of MoleMap NZ Limited and e-derm consult GmbH and undertakes regular teledermatological reporting for both companies. HS is a Medical Consultant for Canfield Scientific Inc, MoleMap Australia Pty Ltd, Blaze Bioscience Inc, Revenio Research Oy, and a Medical Advisor for First Derm. The remaining authors declare that the research was conducted in the absence of any commercial or financial relationships that could be construed as a potential conflict of interest.

## Publisher's Note

All claims expressed in this article are solely those of the authors and do not necessarily represent those of their affiliated organizations, or those of the publisher, the editors and the reviewers. Any product that may be evaluated in this article, or claim that may be made by its manufacturer, is not guaranteed or endorsed by the publisher.

## References

[1] Dinnes J Deeks JJ Grainge MJ Chuchu N. Ferrante di Ruffano L Matin RN. Visual inspection for diagnosing cutaneous melanoma in adults. Cochrane Database Syst Rev. (2018) 12:Cd013194. 10.1002/14651858.CD013194 30521684 PMC6492463

[2] Lee KJ di Meo N Yelamos O Malvehy J Zalaudek I. Soyer HP. Dermoscopy/Confocal Microscopy. In: Balch CM. editors. *Cutaneous Melanoma 6th ed.* Cham: Springer (2019). 10.1007/978-3-319-46029-1_50-2

[3] Moloney FJ Guitera P Coates E Haass NK Ho K Khoury R. Detection of primary melanoma in individuals at extreme high risk: a prospective 5-year follow-up study. JAMA Dermatol. (2014) 150:819–27. 10.1001/jamadermatol.2014.514 24964862

[4] Rayner JE Laino AM Nufer KL Adams L Raphael AP Menzies SW. Clinical perspective of 3D total body photography for early detection and screening of melanoma. Front Med. (2018) 5:152. 10.3389/fmed.2018.00152 29911103 PMC5992425

[5] Welch HG Mazer BL Adamson AS. The rapid rise in cutaneous melanoma diagnoses. N Engl J Med. (2021) 384:72–9. 10.1056/NEJMsb2019760 33406334

[6] Elliott TM Whiteman DC Olsen CM Gordon LG. Estimated healthcare costs of melanoma in Australia over 3 years post-diagnosis. Appl Health Econ Health Policy. (2017) 15:805–16. 10.1007/s40258-017-0341-y 28756584

[7] Pavlick A Weber J. Managing Checkpoint Inhibitor Symptoms and Toxicity for Metastatic Melanoma. In: Balch CM Atkins MB Garbe C. (eds) Cutaneous Melanoma. Cham: Springer International Publishing, (2020). pp.1187-1214. 10.1007/978-3-030-05070-2_60

[8] Spinks J Janda M Soyer HP Whitty JA. Consumer preferences for teledermoscopy screening to detect melanoma early. J Telemed Telecare. (2015) 22:39–46. 10.1177/1357633X15586701 26026184

[9] Welch HG Black WC. Overdiagnosis in cancer. J Natl Cancer Inst. (2010) 102:605–13. 10.1093/jnci/djq099 20413742

[10] Glasziou PP Jones MA Pathirana T Barratt AL Bell KJ. Estimating the magnitude of cancer overdiagnosis in Australia. Med J Aust. (2020) 212:163–8. 10.5694/mja2.50455 31858624 PMC7065073

[11] Bell KJL Mehta Y Turner RM Morton RL Dieng M Saw R. Fear of new or recurrent melanoma after treatment for localised melanoma. Psychooncology. (2017) 26:1784–91. 10.1002/pon.4366 28052599

[12] Ferris LK. Early Detection of Melanoma: Rethinking the Outcomes That Matter. JAMA Dermatol. (2021) 157:511–3. 10.1001/jamadermatol.2020.5650 33729450

[13] Janda M Cust AE Neale RE Aitken JF Baade PD Green AC. Early detection of melanoma: a consensus report from the Australian Skin and Skin Cancer Research Centre Melanoma Screening Summit. Aust N Z J Public Health. (2020) 44:111–5. 10.1111/1753-6405.12972 32190955

[14] Kvaskoff M Siskind V Green AC. Risk factors for lentigo maligna melanoma compared with superficial spreading melanoma: a case-control study in Australia. Arch Dermatol. (2012) 148:164–70. 10.1001/archdermatol.2011.291 22004881

[15] Rayner JE Duffy DL Smit DJ Jagirdar K Lee KJ De'Ambrosis B. Germline and somatic albinism variants in amelanotic/hypomelanotic melanoma: increased carriage of TYR and OCA2 variants. PLoS ONE. (2020) 15:e0238529. 10.1371/journal.pone.0238529 32966289 PMC7510969

[16] Ruijs MW Verhoef S Rookus MA Pruntel R van der Hout AH Hogervorst FB. TP53 germline mutation testing in 180 families suspected of Li-Fraumeni syndrome: mutation detection rate and relative frequency of cancers in different familial phenotypes. J Med Genet. (2010) 47:421–8. 10.1136/jmg.2009.073429 20522432

[17] Butrón-Bris B Daudén E Rodríguez-Jiménez P. Psoriasis therapy and skin cancer: a review. Life (Basel). (2021) 11. 10.3390/life11101109 34685480 PMC8538945

[18] Collett D Mumford L Banner NR Neuberger J Watson C. Comparison of the incidence of malignancy in recipients of different types of organ: a UK Registry audit. Am J Transplant. (2010) 10:1889–96. 10.1111/j.1600-6143.2010.03181.x 20659094

[19] Primiero CA McInerney-Leo AM Betz-Stablein B Whiteman DC Gordon L Caffery L. Evaluation of the efficacy of 3D total-body photography with sequential digital dermoscopy in a high-risk melanoma cohort: protocol for a randomised controlled trial. BMJ Open. (2019) 9:e032969. 10.1136/bmjopen-2019-032969 31712348 PMC6858160

[20] Jackson C Maibach H. Ethnic and socioeconomic disparities in dermatology. J Dermatolog Treat. (2016) 27:290–1. 10.3109/09546634.2015.1101409 26418077

[21] Ferguson NN. Challenges and Controversy in Determining UV Exposure as a Risk Factor for Cutaneous Melanoma in Skin of Color. JAMA Dermatol. (2021) 157:151–3. 10.1001/jamadermatol.2020.4615 33326000

[22] Campiche R Trevisan S Séroul P Rawlings AV Adnet C Imfeld D Voegeli R. Appearance of aging signs in differently pigmented facial skin by a novel imaging system. J Cosmet Dermatol. (2019) 18:614–27. 10.1111/jocd.12806 30381859 PMC7379553

[23] Gallagher RP McLean DI. The epidemiology of acquired melanocytic nevi A brief review. Dermatol Clin. (1995) 13:595–603. 10.1016/S0733-8635(18)30065-2 7554507

[24] Lawson DD Moore DH 2nd Schneider JS Sagebiel RW. Nevus counting as a risk factor for melanoma: comparison of self-count with count by physician. J Am Acad Dermatol. (1994) 31:438–44. 10.1016/S0190-9622(94)70207-1 8077469

[25] Betz-Stablein B Koh U Plasmeijer EI Janda M Aitken JF Soyer HP Green AC. Self-reported naevus density may lead to misclassification of melanoma risk. Br J Dermatol. (2020) 182:1488–90. 10.1111/bjd.18802 31833052

[26] Betz-Stablein B D'Alessandro B Koh U Plasmeijer E Janda M Menzies SW. Reproducible naevus counts using 3D total body photography and convolutional neural networks. Dermatology. (2021) 1–8. 10.1159/000517218 34237739

[27] Betz-Stablein B Llewellyn S Bearzi P Grochulska K Rutjes C Aitken JF. High variability in anatomic patterns of cutaneous photodamage: a population-based study. Journal of the European Academy of Dermatology and Venereology: JEADV. (2021) 35:1896–903. 10.1111/jdv.17352 33991136

[28] Gandini S Sera F Cattaruzza MS Pasquini P Zanetti R Masini C. Meta-analysis of risk factors for cutaneous melanoma: III. Family history, actinic damage and phenotypic factors. Eur J Cancer. (2005) 41:2040–59. 10.1016/j.ejca.2005.03.034 16125929

[29] Shekar SN Duffy DL Youl P Baxter AJ Kvaskoff M Whiteman DC. A population-based study of Australian twins with melanoma suggests a strong genetic contribution to liability. J Invest Dermatol. (2009) 129:2211–9. 10.1038/jid.2009.48 19357710 PMC3672052

[30] Ford D Bliss JM Swerdlow AJ Armstrong BK Franceschi S Green A. Risk of cutaneous melanoma associated with a family history of the disease. The International Melanoma Analysis Group (IMAGE). Int J Cancer. (1995) 62:377–81. 10.1002/ijc.2910620403 7635561

[31] Potrony M Badenas C Aguilera P Puig-Butille JA Carrera C Malvehy. Update in genetic susceptibility in melanoma. Ann Transl Med. (2015) 3:210. 10.3978/j.issn.2305-5839.2015.08.11 26488006 PMC4583600

[32] Aoude LG Heitzer E Johansson P Gartside M Wadt K Pritchard AL. POLE mutations in families predisposed to cutaneous melanoma. Fam Cancer. (2015) 14:621–8. 10.1007/s10689-015-9826-8 26251183

[33] Moran A O'Hara C Khan S Shack L Woodward E Maher ER. Risk of cancer other than breast or ovarian in individuals with BRCA1 and BRCA2 mutations. Fam Cancer. (2012) 11:235–42. 10.1007/s10689-011-9506-2 22187320

[34] Bertolotto C Lesueur F Giuliano S Strub T de Lichy M Bille K. A SUMOylation-defective MITF germline mutation predisposes to melanoma and renal carcinoma. Nature. (2011) 480:94–8. 10.1038/nature10539 22012259

[35] Cust AE Harland M Makalic E Schmidt D Dowty JG Aitken JF. Melanoma risk for CDKN2A mutation carriers who are relatives of population-based case carriers in Australia and the UK. J Med Genet. (2011) 48:266–72. 10.1136/jmg.2010.086538 21325014 PMC7432952

[36] Box NF Duffy DL Chen W Stark M Martin NG Sturm RA Hayward NK. MC1R genotype modifies risk of melanoma in families segregating CDKN2A mutations. Am J Hum Genet. (2001) 69:765–73. 10.1086/323412 11500805 PMC1226062

[37] Primiero CA Yanes T Finnane A Soyer HP McInerney-Leo AM. A Systematic Review on the Impact of Genetic Testing for Familial Melanoma II: Psychosocial Outcomes and Attitudes. Dermatol. (2021) 237:816–26. 10.1159/000513576 33508831

[38] Primiero CA Yanes T Finnane A Soyer HP McInerney-Leo AM. A systematic review on the impact of genetic testing for familial melanoma I: primary and secondary preventative behaviours. Dermatol. (2021) 237:806–15. 10.1159/000513919 33588421

[39] Landi MT Bishop DT MacGregor S Machiela MJ Stratigos AJ Ghiorzo P. Genome-wide association meta-analyses combining multiple risk phenotypes provide insights into the genetic architecture of cutaneous melanoma susceptibility. Nat Genet. (2020) 52:494–504. 10.1038/s41588-020-0611-8 32341527 PMC7255059

[40] Yanes T McInerney-Leo AM Law MH Cummings S. The emerging field of polygenic risk scores and perspective for use in clinical care. Hum Mol Genet. (2020) 29:R165–r176. 10.1093/hmg/ddaa136 32620971

[41] Yanes T Willis AM Meiser B Tucker KM Best M. Psychosocial and behavioral outcomes of genomic testing in cancer: a systematic review. Eur J Hum Genet. (2019) 27:28–35. 10.1038/s41431-018-0257-5 30206354 PMC6303287

[42] Smit AK Allen M Beswick B Butow P Dawkins H Dobbinson SJ. Impact of personal genomic risk information on melanoma prevention behaviors and psychological outcomes: a randomized controlled trial. Genet Med. (2021). 10.1038/s41436-021-01292-w 34385669 PMC8629758

[43] Esteva A Kuprel B Novoa RA Ko J Swetter SM Blau HM. Thrun S. Dermatologist-level classification of skin cancer with deep neural networks. Nature. (2017) 542:115–8. 10.1038/nature21056 28117445 PMC8382232

[44] Tschandl P Codella N Akay BN Argenziano G Braun RP Cabo H. Comparison of the accuracy of human readers versus machine-learning algorithms for pigmented skin lesion classification: an open, web-based, international, diagnostic study. Lancet Oncol. (2019) 20:938–47. 10.1016/S1470-2045(19)30333-X 31201137 PMC8237239

[45] Tschandl P Rinner C Apalla Z Argenziano G Codella N Halpern A. Human-computer collaboration for skin cancer recognition. Nat Med. (2020) 26:1229–34. 10.1038/s41591-020-0942-0 32572267

[46] Rotemberg V Kurtansky N Betz-Stablein B Caffery L Chousakos E Codella N. A Patient-Centric Dataset of Images and Metadata for Identifying Melanomas Using Clinical Context. Sci Data. (2021) 28:34–41. 10.1038/s41597-021-00815-z 33510154 PMC7843971

[47] Yu Z Nguyen J Nguyen TD Kelly J Mclean C Bonnington P. Early Melanoma Diagnosis with Sequential Dermoscopic Images. IEEE Trans Med Imaging. (2021). 10.1109/TMI.2021.3120091 34648437

[48] Soenksen LR Kassis T Conover ST Marti-Fuster B Birkenfeld JS Tucker-Schwartz J. Using deep learning for dermatologist-level detection of suspicious pigmented skin lesions from wide-field images. Sci Transl Med. (2021) 13. 10.1126/scitranslmed.abb3652 33597262

[49] Lin LL Prow TW Raphael AP Harrold Iii RL Primiero CA Ansaldo AB Soyer HP. Microbiopsy engineered for minimally invasive and suture-free sub-millimetre skin sampling. F1000Res. (2013) 2:120. 10.12688/f1000research.2-120.v1 24627782 PMC3907159

[50] Tan JM Tom LN Jagirdar K Lambie D Schaider H Sturm RA. The BRAF and NRAS mutation prevalence in dermoscopic subtypes of acquired naevi reveals constitutive mitogen-activated protein kinase pathway activation. Br J Dermatol. (2018) 178:191–7. 10.1111/bjd.16205 28714107

[51] Grossman D Okwundu N Bartlett EK Marchetti MA Othus M Coit DG. Prognostic Gene Expression Profiling in Cutaneous Melanoma: Identifying the Knowledge Gaps and Assessing the Clinical Benefit. JAMA Dermatology. (2020) 156:1004–11. 10.1001/jamadermatol.2020.1729 32725204 PMC8275355

[52] Schadendorf D Hauschild A Santinami M Atkinson V Mandalà M Chiarion-Sileni V. Patient-reported outcomes in patients with resected, high-risk melanoma with BRAF(V600E) or BRAF(V600K) mutations treated with adjuvant dabrafenib plus trametinib (COMBI-AD): a randomised, placebo-controlled, phase 3 trial. Lancet Oncol. (2019) 20:701–10. 10.1016/S1470-2045(18)30940-9 30928620

[53] Yarchoan M Hopkins A. Jaffee EM. Tumor Mutational Burden and Response Rate to PD-1 Inhibition. N Engl J Med. (2017) 377:2500–1. 10.1056/NEJMc1713444 29262275 PMC6549688

[54] Ganini C Amelio I Bertolo R Bove P Buonomo OC Candi E. Global mapping of cancers: The Cancer Genome Atlas and beyond. Mol Oncol. (2021) 15:2823–40. 10.1002/1878-0261.13056 34245122 PMC8564642

[55] Binder H Schmidt M Loeffler-Wirth H Mortensen LS Kunz M. Melanoma single-cell biology in experimental and clinical settings. J Clini Med. (2021) 10. 10.3390/jcm10030506 33535416 PMC7867095

[56] Wan Q Liu C Liu C Liu W Wang X Wang Z. Discovery and validation of a metastasis-related prognostic and diagnostic biomarker for melanoma based on single cell and gene expression datasets. Front Oncol. (2020) 10:585980. 10.3389/fonc.2020.585980 33324561 PMC7722782

[57] Merritt CR Ong GT Church SE Barker K Danaher P Geiss G. Multiplex digital spatial profiling of proteins and RNA in fixed tissue. Nat Biotechnol. (2020) 38:586–99. 10.1038/s41587-020-0472-9 32393914

[58] Ståhl PL Salmén F Vickovic S Lundmark A Navarro JF Magnusson J. Visualization and analysis of gene expression in tissue sections by spatial transcriptomics. Science. (2016) 353:78–82. 10.1126/science.aaf2403 27365449

[59] Snoswell CL Whitty JA Caffery LJ Kho J Horsham C Loescher LJ. Consumer preference and willingness to pay for direct-to-consumer mobile teledermoscopy services in Australia. Dermatol. (2021) 1–10. 10.1159/000517257 34515087 PMC8985042

[60] Rat C Hild S Rault Sérandour J Gaultier A Quereux G Dreno B Nguyen JM. Use of smartphones for early detection of melanoma: systematic review. J Med Internet Res. (2018) 20:e135. 10.2196/jmir.9392 29653918 PMC5923035

[61] Koh U Horsham C Soyer HP Loescher LJ Gillespie N Vagenas D Janda M. Consumer acceptance and expectations of a mobile health application to photograph skin lesions for early detection of Melanoma. Dermatology. (2019) 235:4–10. 10.1159/000493728 30404081

[62] Weinstock MA Nguyen FQ Risica PM. Patient and referring provider satisfaction with teledermatology. J Am Acad Dermatol. (2002) 47:68–72. 10.1067/mjd.2002.119666 12077584

[63] Horsham C Snoswell C Vagenas D Loescher LJ Gillespie N Soyer HP Janda M. Is teledermoscopy ready to replace face-to-face examinations for the early detection of skin cancer? Consumer views, technology acceptance, and satisfaction with care. Dermatology. (2020) 236:90–6. 10.1159/000506154 32114570

[64] Gillespie N Lockey S Curtis C. Trust in Artificial Intelligence: A Five Country Study. (2021). The University of Queensland and KPMG Australia. 10.14264/e34bfa3

[65] Scheetz J Rothschild P McGuinness M Hadoux X Soyer HP Janda M. A survey of clinicians on the use of artificial intelligence in ophthalmology, dermatology, radiology and radiation oncology. Sci Rep. (2021) 11:5193. 10.1038/s41598-021-84698-5 33664367 PMC7933437

[66] Gomolin A Netchiporouk E Gniadecki R Litvinov IV. Artificial intelligence applications in dermatology: where do we stand? Front Med. (2020) 7. 10.3389/fmed.2020.00100 32296706 PMC7136423

[67] Digital Imaging and Communications in Medicine. WG-19 Dermatology. (2021) Available online at: https://www.dicomstandard.org/activity/wgs/wg-19 (accessed 26 December, 2021).

[68] Caffery LJ Clunie D Curiel-Lewandrowski C Malvehy J Soyer HP Halpern AC. Transforming dermatologic imaging for the digital era: metadata and standards. J Digital Imaging. (2018) 31:568–77. 10.1007/s10278-017-0045-8 29344752 PMC6113154

[69] Supplement 221: Dermoscopy. Available online at: https://www.dicomstandard.org/News/current/docs/sups/sup221.pdf (accessed 26 December, 2021)

[70] Caffery LJ Rotemberg V Weber J Soyer HP Malvehy J Clunie D. The role of DICOM in artificial intelligence for skin disease. Front Med. (2021) 7:1163. 10.3389/fmed.2020.619787 33644087 PMC7902872

